# Expression profiles of hydrophobic surfactant proteins in children with diffuse chronic lung disease

**DOI:** 10.1186/1465-9921-6-80

**Published:** 2005-07-22

**Authors:** Matthias Griese, Silja Schumacher, Mohammed Tredano, Manuela Steinecker, Annika Braun, Susan Guttentag, Michael F Beers, Michel Bahuau

**Affiliations:** 1 Kinderklinik and Poliklinik, Dr. von Haunersches Kinderspital, Ludwig-Maximilians University, Munich, Germany; 2 Service de Biochimie et Biologie Moléculaire, Hôpital d'Enfants Armand-Trousseau (AP-HP), Paris, France; 3 Division of Neonatology, Childrens' Hospital of Philadelphia, University of Pennsylvania School of Medicine, Philadelphia, Pennsylvania 19104-4318, USA; 4 Pulmonary and Critical Care Division, University of Pennsylvania School of Medicine Philadelphia, Pennsylvania 19104-6160, USA

**Keywords:** *SFTPB*, *SFTPC*, SP-B deficiency, SP-C, pro-SP-C, processing, pulmonary alveolar proteinosis (PAP), unexplained respiratory distress, interstitial lung disease, children, infant, neonate

## Abstract

**Background:**

Abnormalities of the intracellular metabolism of the hydrophobic surfactant proteins SP-B and SP-C and their precursors may be causally linked to chronic childhood diffuse lung diseases. The profile of these proteins in the alveolar space is unknown in such subjects.

**Methods:**

We analyzed bronchoalveolar lavage fluid by Western blotting for SP-B, SP-C and their proforms in children with pulmonary alveolar proteinosis (PAP, n = 15), children with no SP-B (n = 6), children with chronic respiratory distress of unknown cause (cRD, n = 7), in comparison to children without lung disease (n = 15) or chronic obstructive bronchitis (n = 19).

**Results:**

Pro-SP-B of 25–26 kD was commonly abundant in all groups of subjects, suggesting that their presence is not of diagnostic value for processing defects. In contrast, pro-SP-B peptides cleaved off during intracellular processing of SP-B and smaller than 19–21 kD, were exclusively found in PAP and cRD. In 4 of 6 children with no SP-B, mutations of *SFTPB *or *SPTPC *genes were found. Pro-SP-C forms were identified at very low frequency. Their presence was clearly, but not exclusively associated with mutations of the *SFTPB *and *SPTPC *genes, impeding their usage as candidates for diagnostic screening.

**Conclusion:**

Immuno-analysis of the hydrophobic surfactant proteins and their precursor forms in bronchoalveolar lavage is minimally invasive and can give valuable clues for the involvement of processing abnormalities in pediatric pulmonary disorders.

## Introduction

Pulmonary surfactant is a highly surface active complex of lipids and specific proteins, including surfactant proteins (SP-) A, B, C and D [1]. The maintenance of the patency of the airspaces at end-expiration is heavily dependent on the phospholipid components and their interaction with SP-B and SP-C [2]. SP-B is encoded by a single gene (*SFTPB*) [3] and translated in the alveolar type II cells into a preproprotein (~40 kDa). Post-translational processing of pro-SP-B to yield mature SP-B is a multistep entirely intracellular process involving multiple sites and enzymes [4-7]. SP-C is encoded by the *SFTPC *gene on chromosome 8 [8] and the SP-C proprotein processing [9-11] is integrally linked to the metabolism of SP-B in that infants and mice with genetic SP-B deficiency exhibit incompletely processed pro-SP-C peptides of 6–14 kDa in intra- and extracellular surfactant [12,13]. In lung homogenates of most infants with *SFTPB *mutations, aberrant pro-SP-C forms (Mr 6–12 kD) are observed [14]. Similarly, pro-SP-B forms of variable sizes have been detected in lung homogenates from some children with chronic lung disease but were predominantly absent in patients with *SFTPB *mutations [14].

Bronchoalveolar lavage (BAL) is a commonly used first line diagnostic tool to sample the alveolar space content and this technique is much less invasive than open lung biopsy. Thus the profiles of SP-B, SP-C and their propeptide precursors present in the extracellular, intraalveolar space represent a potential diagnostic tool for assessment of neonatal and childhood lung disease.

Neonates with respiratory distress of unknown cause are likely candidates for abnormalities of SP-B and SP-C metabolism. Similarly, but much less appreciated, SP-B and SP-C abnormalities might play a role in infants or older children with chronic respiratory distress developing beyond the neonatal period. Pediatric pulmonary alveolar proteinosis (PAP) is a rare abnormality of the surfactant metabolism, characterized by the accumulation of large amounts of surfactant in the alveolar space, leading to gas exchange abnormalities [15,16]. In contrast to the adult form of acquired PAP where GM-CSF autoantibodies appear to play a pathogenic role, the causes of pediatric PAP are as yet unresolved. In particular the characteristics of SP-B and SP-C peptides and their precursors in the alveolar space of pediatric patients with lung disease have not been described.

Using defined pediatric patient populations, Western blotting of BAL identified several distinct banding profiles for the hydrophobic surfactant proteins and their precursors. These data support the feasibility of using immunoanalyses of BAL fluid to evaluate chronic pediatric pulmonary disorders in more detail.

## Patients, Materials and methods

### Patients

The lavage effluents from 15 children without lung disease and 19 children with chronic obstructive bronchitis were used as controls or disease controls, for comparison with the lavage effluents that were available from our previously described cohort of neonatal, pediatric or juvenile patients with respiratory distress of unknown cause. These children were seen in western European medical hospital centers (mainly from France and Germany) and were analyzed for a genetic defect leading to deficiency in SP-B and SP-C [17,18].

The lavage effluents from the children without lung disease were aliquots obtained previously in a study that assessed inflammation in children with chronic tracheostoma in comparison to these controls [19]. The lavage effluents were obtained during anesthesia for elective surgery for minor conditions. The usage of this material and that of the children with chronic bronchitis for this study was approved by the ethics committee at the University of Munich. Written informed consent was obtained from the patients where appropriate from age and from the caregivers.

Children with chronic obstructive bronchitis in whom anomalies of the airways, cystic fibrosis, primary ciliary dyskinesia, gastro-esophageal reflux, immuno deficiencies, allergic asthma and passive smoke exposure were excluded as causes and in whom a lavage was performed during the diagnostic work up, were used as a disease control group. The obstruction was determined by chest auscultation during the course of the disease. Details of these patients are given in table 1.

**Table 1:** Patient characteristics, lavage protein content and apparent molecular weight of SP-B and SP-C

Children	N (males)	Age (y)	Time of follow up (years), outcome	Protein (μg/ml)	SP-B M_r _of band (kDa)	SP-C M_r _of band (kDa)
without lung disease	15 (8)	5.4 (0.5–12)	not applicable	62 (21–275)	7.1 (5.9–11.6)	4.8 (4.3–5.8)
with chronic obstructive bronchitis	19 (13)	5.3 (1–15)	4 (0.3–10) years, 14/19 better, 3/19 same, 1/19 worse, 1/19 unknown	76 (17–207)	11.0 (8–13.5)	5.2 (3.9–5.6)
with no SP-B	6 (3)	neonates	5 pts [2–6] died at 0.3 (0.1–0.4) years, pt [1] alive with corticosteroids	318* (131–2048)	no SP-B bands in any pt	5.6 (3.6–6.5) pt [4] no SP-C
with pulmonary alveolar proteinosis	15 (9)	1.4 (0.6–4)	Pts [6,10,14,15] died at ages 1.3 and 1.7 years and at 4 and 5 months of age. 11/15 alive with repetitive whole lung lavages and oxygen-dependence	495** (87–2099)	10.5 (8.8–12.5)	4.8 (3.6–5.4)
with chronic respiratory distress of unknown cause	7 (7)	neonates, one subject 4 months	4 died at age 8 days to 4 months, 3 [3,6,7] lost on follow up	449* (184–474)	9.7 (6.3–11.2)	5.6 (4.3–7)

All data are medians and range, n.d. = not determined. Significantly higher compared to children without lung disease or children with obstructive bronchitis, which did not differ *p < 0.01, **p < 0.001 by Kruskal-Wallis-Analysis followed by Dunn's multiple comparisons test

From the cases with SP-B deficiency we initially described, sufficient BAL material for analysis was available from 6 neonates (URD 6-II.1, 2-II.1, 7-II.1, 4-II.1, 3-II.1, 9-II.4), now labeled no-SP-B 1–6. All these babies had respiratory distress, and alveolar infiltrates with various degrees of interstitial involvement. A congenital heart disease or a lung disease due to mycoplasma, chlamydia, and viruses had also been ruled out. Details on the subjects are given in table 1. All but 2 subjects had mutations of SP-B as the cause for the SP-B deficiency.

From the cases with pulmonary alveolar proteinosis, sufficient BAL material for analysis was available from 15 children (URD 10-II.1, 11-II.3, 17-II.2, 25-II.3, 19-II.1, 20-II.2, 21-II.1, 16-II.2, 27-II.3, 22-II.1, 26-II.1, 23-II.3, 13-II.1, 13-II.2, 18-II.2), now labeled PAP 1–15. Most of these cases were less severely affected, had dyspnea and progressive cough, sometimes accompanied by cyanosis, finger clubbing, failure to thrive in the younger ones, and asthenia or weight loss in the others. Chest x-ray showed typical alveolar as well as interstitial infiltrates (table 1). In all these patients mutations of SP-B were excluded, 3 patients (PAP 04, PAP 10 and PAP 12) had heterozygous mutations in SFTPC. None of these children was investigated for ABCA3 mutations. All known secondary causes of PAP were excluded.

In addition, 7 subjects with chronic respiratory distress of unknown cause, in the absence of SP-B deficiency or alveolar proteinosis were investigated. BAL was available from 6 (URD 31-II.3, 40-II.1, 36-II.2, 30-II.1, 39-II.1, 37-II.2) of the initial 15 patients and from another infant born at 36 wks of gestation, with acute respiratory distress and development of chronic respiratory distress of unknown cause, after exclusion of SP-B, SP-C deficiency, and pulmonary alveolar proteinosis. None of these children was investigated for ABCA3 mutations. The children were labeled cRD 1–7 and their outcomes are given in table 1.

### Bronchoalveolar lavage

Routinely, the fluid recovered from BAL (4 × 1 ml 0.9% NaCl/kg body weight, b.w.) was pooled and the cells separated before analysis. Alternatively, in very sick neonates, repetitive tracheal aspirates after the instillation of 1 ml 0.9%NaCl/kg b.w. were collected over time periods of several hours up to a week, pooled and used for biochemical analyses.

### Antisera

**Figure 1:**
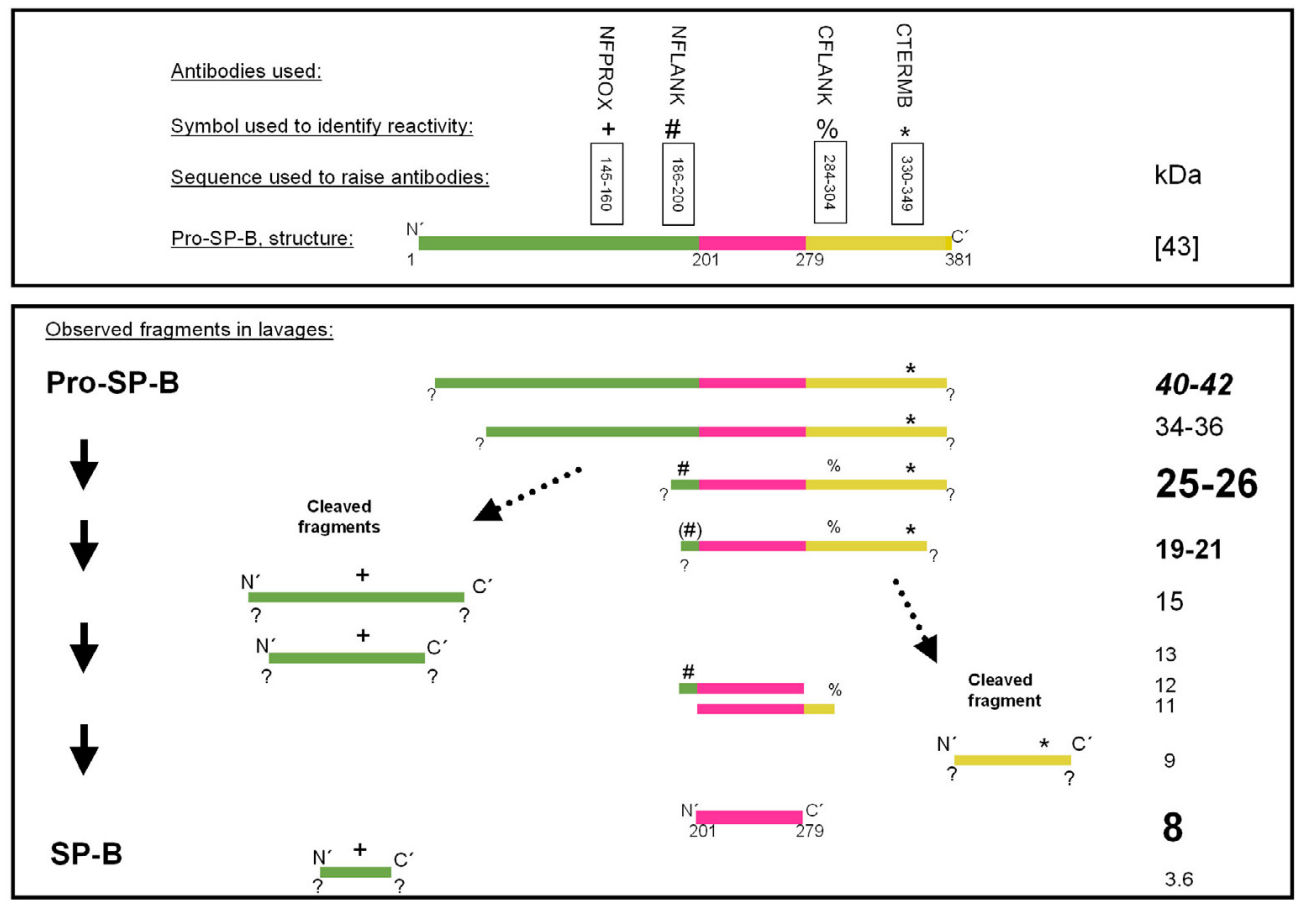
Schematic diagram of pro-SP-B and its processing to SP-B. Upper panel: Indicated are the antibodies used, the symbols for their identification, the amino acid stretches against which the antibodies were developed, and a diagram of the structure of pro-SP-B. Lower panel: The molecular weight and the reactivity of the antibodies (in the absence, but not in the presence of the competing peptides) during Western blotting is indicated. The sizing of the letters used for indication of the molecular weights is proportional to the frequency at which the bands were observed (biggest: common >75% of subjects, 2^nd ^biggest: frequent, in <75 but >50% of the subjects, 3^rd ^biggest: sporadic, in <50 but >25% of the subjects, smallest: rare, in <25% of the subjects). The sequence of SP-B within the pro-SP-B sequence is indicated in pink. All bands were analyzed under reducing conditions.

All antisera used were polyclonal and raised in rabbits. The antibodies against SP-B (c329) and SP-C (22/96) were gifts from Dr W. Steinhilber, Altana AG, Konstanz, FRG and were used at a dilution of 1:10,000 [20]. The antisera against pro-SP-B were raised against peptides of pro-SP-B, which were also used to determine the specificity of the signals on the immunoblots in all cases. The abbreviations and location of these peptides in the pro-SP-B sequence is indicated in figure 1. NFPROX was raised against SRQPEPEQEPGMSDPL, NFLANK against QARPGPHTQDLSEQQ, both were used at 1:2000 dilution. CFLANK was raised against GPRSPTGEWLPRDSECHLCMS, used at 1:1000 dilution and CTERMB was raised against LDREKCKQFVEQHTPQLLTL, used at 1:5000 dilution. Pro-SP-C was detected by anti-serum used at 1:5.000 dilution and raised previously against ESPPDYSTGPRSQ, i.e. Glu10–Gln23 of the amino acid sequence in pro-SP-C. The characteristics of all these antibodies has been described previously in detail [21-23].

### Surfactant protein characterization

Total protein content of the samples was determined with the Biorad Protein Assay Kit (Biorad, Richmond, CA), which is based on the method by Bradford [24]. Ten to twenty-five μg of total protein were separated under reducing conditions on NuPage10% Bis-Tris gels using a NOVEX X-cell II Mini-Cell system (Novex, San Diego, CA). At least two sets of gels were prepared in parallel for each patient. Following electrophoresis the gels were either silver stained [25], or subjected to Western transfer. For immunodetection, the proteins in the gels were transfered onto a PVDF membrane (ImmobilonP, Millipore, Bedford, MA) with a NuPage Blot module (Novex, San Diego, CA) according to the manufacturers recommendations.

Surfactant proteins and their pro-forms were detected on the PVDF membrane by immunoblot using the polyclonal rabbit antisera described in detail above, and the enhanced chemiluminescence assay (Amersham Biosciences, Buckinghamshire, UK) with horseradish peroxidase conjugated goat anti-rabbit polyclonal anti-IgG (1:10,000; Dianova, Hamburg, FRG).

To verify the specificity of the antibodies used to probe the pro-forms of SP-B and SP-C, a duplicate blot was prepared in each case and probed with an antibody solution containing 1 μM of the peptide, against which the antibody was raised. Antigen specific bands on the blot disappeared under these conditions. The blots were developed by exposure of X-ray film (Hyperfilm ECL, Amersham Biosciences, Buckinghamshire, UK) to the blots.

In the group of controls blots were first incubated with antibody against CTERMB and after that with the SP-B antibody respectively first with antibody against SP-C and after that with the pro-SP-C antibody, with and without competing peptide. In the other groups there were separate blots for each incubation with antibodies against SP-B and SP-C and their proforms, with and without competing peptide. Under these conditions the assay could detect about 2.5 ng of SP-B or SP-C per lane. In several experiments, aliquots of a patient with pro-SP-C forms were run in parallel as a positive control for pro-SP-C forms.

Immunoblots and silver stained gels were scanned with the Fluor-S MultiImager (Biorad, Richmond, CA) gel documentation system, and the resulting images were analyzed with the Software MultiAnalyst (Biorad, Richmond, CA).

### Deglycosylation

To determine if the proteins that reacted with the CTERMB antibody on the immunoblots were glycosylated, the samples were deglycosylated before applying them on the gel (4). In brief, 1 unit of recombinant N-glycosidase F (Roche Molecular Biochemicals, Mannheim) was added to 500 μl incubation buffer (100 mM Na-phosphate, 25 mM EDTA, 1% β-mercaptoethanol, 0.5% Triton X-100, 0,1% SDS, pH 7.2). The vacuum dried sample was resuspended in 20 μl of this solution and incubated for 15 h at 37°C. The sample was then vacuum dried and analyzed by Western immunoblot.

### Genetic analysis

For *SFTPB *mutation screening, first the 121ins2 frame-shift mutation was searched using the restriction enzyme cleavage *Sfu*I endonuclease by PCR. In 121ins2-negative patients, *SFTPB *exons 1–11 and the promoter region were PCR-amplified and the purified PCR products served as templates in the sequencing reaction using Ready Reaction Dye Terminator Cycle Sequencing Kit With AmpliTaq^® ^DNA Polymerase, FS (PEBiosystems, Foster City, CA) with forward and reverse PCR oligonucleotides used as extension primers. Extension products were analyzed using the ABIPRISM™ 310 Genetic analysis System (PEBiosystems), as previously reported in detail [18]. Similarly, *SFTPC *exons 1–6 were analysed [17].

### Statistical analysis

Statistical calculations were performed with the Software GraphPad Prism 4.0 (GraphPad Software, San Diego, CA). Differences in nonparametric values were calculated with the Kruskal-Wallis test. For pair wise comparisons of groups we used Dunn's test (2). Differences in frequencies were calculated with the Fisher exact test. Correlation coefficients were determined according to Pearson. Results with a p ≤ 0.05 were considered significant.

## Results

### Children without lung disease and children with chronic bronchitis

The children with chronic obstructive bronchitis had a slight increase in neutrophils (3% (2; 15)(data are median and (25.; 75. percentile)) compared to children without lung disease (1% (1; 2); p = 0.035) and a somewhat lower viability (80% (70; 90) and 90% (80; 97) in children without lung disease; p = 0,035). The other variables did not differ and were within the normal range, i.e. children with chronic obstructive bronchitis: total cell count 150/μl (82; 275), macrophages 80% (69; 90) of total cells, lymphocytes 10% (4/14), eosinophils 0% (0; 2) and recovery was 54% (39; 70) and the children without lung disease: total cell count 115/μl (82; 180), macrophages 87% (82; 92) of total cells, lymphocytes 11.5% (7; 14.5), eosinophils 0% (0; 0.5) and recovery was 48% (42; 62).

**Figure 2:**
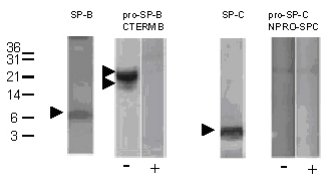
Children with chronic bronchitis. Representative Western blotting pattern of BAL from child with chronic bronchitis (patient control 03). After SDS-PAGE and transfer, the membranes were probed with different antibodies directed against SP-B, certain sequences of the pro-SP-B, in the absences (-) and presence (+) of excess of the peptides, used to raise the antibodies, SP-C and against pro-SP-C, in the absence (-) and presence (+) of excess of the N-terminal peptide, used to raise these antibodies. The numbers next to the lanes indicate the molecular weight in kDa. The arrow heads indicate bands of interest, as described in the text. All bands were analyzed under reducing conditions.

Mature SP-B was regularly detected in all lavages from normal children and from those with chronic bronchitis at a median molecular weight of 7 kDa (Tab. 1, Fig. 2). Similarly, pro-SP-B forms with a molecular weight of 25–26 kDa were commonly observed using an antibody against the C-terminal flanking propeptide of pro-SP-B (Tab. 2, Fig. 2). Those bands never reacted with NFPROX, but showed reactivity with NFLANK, demonstrating that this was a processing intermediate generated by removal of the proximal N-terminal amino acids. A similar, but somewhat more truncated, 19–21 kDa pro-SP-B fragment was detected sporadically in these children (Tab. 2, Fig. 2). The pro-SP-B forms at 25–26 and 19–21 kDa were glycosylated as treatment with N-glycosidase F resulted in a significant drop in size for both peptides (not shown). A 40–42 kDa form and a 34–36 kDa form of pro-SP-B were rarely detected. Except for a single case when a 9 kDa C-terminal cleavage fragment was observed, in these children no other cleavage products of pro-SP-B processing were identified. Mature SP-C with M_r _of 5.0 kDa was present in both controls and children with chronic bronchitis, whereas pro-SP-C forms were never detected in BAL (Tabs. 1 and 2, Fig. 2).

**Table 2:** Pro-SP-B and pro-SP-C in the comparison groups, i.e. children without lung disease and in children with chronic bronchitis.

	pro-SP-B							pro-SP-C
Detecting antibody	CTERMB				CFLANK	NFLANK	NFPROX	
M_r _of band	40–42	34–36	25–26	19–21	9	25–26		
Children without lung disease (n = 15)	7% [8]	0%	80% [1,3,4,6–8,10–15]	7% [3]	nd	nd	nd	no bands
Chronic obstructive bronchitis (n = 19)	5% [14]	26% [12–14,18]	100% [1–19]	37% [4–6,10,13,15,18]	5% [**15**]	21% [4–6,9]	no bands	no bands

Percent of subjects with bands and identification numbers of those subjects in whom bands reacting with the anti-pro-SP-B-antibodies CTERMB, NFLANK, CFLANK and NFPROX displaced by the CTERMB, CFLANK, NFLANK or NFPROX peptides, or the anti-pro-SP-C-antibody NPRO-SP-C-C2 and displaced by the respective peptide, were identified. The identification numbers of the patients are given in square brackets []. Numbers in bold refers to bands not identified by the CTERMB antibodies. Due to shortage of lavage material in the normal controls (no lung disease), not all 4 antibodies were tested in this group (nd = not done).

### Children with no SP-B

**Figure 3:**
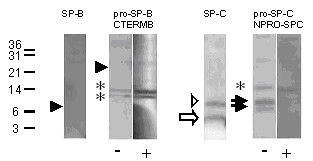
SP-B deficiency. Western blotting of a lavage from patient SP-B 06 homozygous for the 121ins2 *SFTPB *mutation. After SDS-PAGE and transfer, the membranes were probed with the antibodies indicated. The pro-forms were probed in the absence (-) and presence (+) of an excess of the peptide used to raise this antibody. Note that bands that are not displaced by the competing peptide were not considered as specific bands (marked by an asterisk). The numbers next to the lanes indicate the molecular weight in kDa. The closed arrowheads indicate the absence of SP-B and of proforms of SP-B. Arrows show the presence of SP-C (open arrow) and of abberant pro-SP-C (closed arrows). Some aberrant pro-SP-C can also be seen on the SP-C blot, above the SP-C band, which is indicated by an open arrowhead. All bands were analyzed under reducing conditions.

6 of all children investigated did not have SP-B in their lavages. Of these, 4 had lethal mutations of the *SFTPB *gene, i.e. SP-B deficiency (Tab. 3). Pro-SP-B processing products were not found in patient 5, having a 457delC/121ins2 compound heterozygote mutation (Fig. 3, Tab. 3). Unexpectedly, patients 3 and 6, homozygous for 121ins2, and patient 2 homozygous for 496delG had small but specific (competitive) pro-SP-B bands at about 19–21, 25–26 or 34–36 kDa (Tab. 3). Aberrant pro-SP-C bands previously thought to be diagnostic of SP-B mutations were only detected in 121ins2-mutations but not with 457delG [17,26] or with 496delG mutations.

**Table 3:** Pro-SP-B and pro-SP-C in children with no SP-B

		pro-SP-B				pro-SP-C
	Detecting antibody	CTERMB			NFLANK	NPROSP-C-C2
	M_r _of band (kDa)	34–36	25–26	19–21	25–26	
Subject	Genetic analysis of *SFTPB*					
no SP-B 01	no *SFTPB *mutation; marker exclusion	-	++	-	-	-
no SP-B 02	496delG homozygote	+	+	+	-	-
no SP-B 03	121ins2 homozygote	-	+	-	-	6 and 7.9 kDa
no SP-B 04	no *SFTPB *mutation	-	++	-	-	-
no SP-B 05	457delC/121ins2 compound heterozygotes	-	-	-	-	-
no SP-B 06	121ins2 homozygote	-	-	-	+	6.6. and 9 kDa

Bands reacting with the anti-pro-SP-B-antibodies CTERMB, NFLANK, CFLANK and NFPROX displaced by the CTERMB, NFLANK, CFLANK or NFPROX peptides are indicated by "+", or the anti-pro-SP-C-antibody NPRO-SP-C-C2 and displaced by the respective peptide are indicated by the molecular weight directly.

In the other two infants with no SP-B in the lavages, *SFTPB *and *SFTPC *mutations were excluded [17,18]. These patients had significant amounts of pro-SP-B at 25–26 kDa, similar to that observed in the comparison groups. They also did not have pro-SP-C forms in their lavages, providing additional indirect evidence against SP-B processing defects. However, one of these two patients, i.e. patient 4 (Tab. 1), also lacked mature SP-C. This infant died at the age of 1 month from respiratory failure. This case suggests the presence of SP-B and SP-C processing defects arising by means other than from mutations of these genes, i.e. alterations in the protein processing machinery or in the lipid transporters, like ABCA3, as recently shown [27]. The other child (patient 1, Tabs. 1 and 3) is still alive with corticosteroids.

### Children with PAP

**Figure 4:**
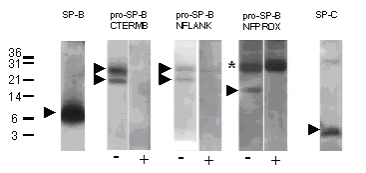
Children with pulmonary alveolar proteinosis. Western blotting of a lavage from patient PAP 12 (only NFPROX bands) and PAP 04 (all other bands) to demonstrate the most frequent abnormalities. After SDS-PAGE and transfer, the membranes were probed with the antibodies indicated. The pro-forms were probed in the absence (-) and presence (+) of an excess of the peptide used to raise this antibody. Note that bands that are not displaced by the competing peptide were not considered as specific bands and they are marked by an asterisk. The numbers next to the lanes indicate the molecular weights in kDa. The arrowheads indicate the abundance of SP-B, the bands at 19–21 and 25–26 kDa using CTERMB which also react with NFLANK, and some of the break-down fragments reacting with NFPROX which are more frequently seen in this condition and in cRD as compared to the other lung diseases (see figure 5). All bands were analyzed under reducing conditions.

In all subjects with PAP, except patient 5, antibodies against GM-CSF in their sera or lavages were excluded in the pathogenesis of their disease. Although SP-B was abundantly present and mutations of *SFTPB *were excluded [18], alterations of SP-B processing from other causes have not been excluded. In general, the same pro-SP-B processing products were observed as in the control and the chronic bronchitis group, however, the 25–26 kDa band was stained by NFLANK at increased frequency (Tab. 4, Fig. 4, lanes 4 and 5). In addition, 15 kDa and 13 kDa bands were present that were only stained by NFPROX. These peptides represent the N-terminal cleaved processing fragments, which were detected only in these patients and not in the respective control group (Tab. 4, Fig. 4, lanes 6 and 7). Three of the PAP patients (PAP 14, PAP 05 and PAP 10) had bands reacting merely with CFLANK or NFLANK. These bands were at 8, 9, 11 and 12 kDa. These may represent imprecisely processed SP-B, still having not completely removed small N- or C-terminal peptide stretches (Figs. 1, Tab. 4).

**Table 4:** Pro-SP-B and pro-SP-C in 15 children with pulmonary alveolar proteinosis

	pro-SP-B				pro-SP-C
Detecting antibody	CTERMB	CFLANK	NFLANK	NFPROX	NPROSP-C-C2
M_r _of bands(kDa)					
40–42	7% [5]	-	-	-	-
34–36	7% [5]	-	-	-	-
25–26	93% [1–5,7–15]	20% [3,5,15]	87%^+ ^[1–5,7–12,14,15]	-	-
19–21	87%* [1–5,7–13,15]	7% [5]	20% [4,5,9]	7% [4]	7% [8]
15	7% [8]	-	7% [8]	33%^+ ^[**2**,8,**9,11,12**]	7% [8]
13	-	-	-	20% [**3,8,9**]	-
12	-	-	7% [**14**]	-	-
11	-	7% [**14**]	-	-	7% [8]
9	-	7% [**10**]	14% [**5,10**]	-	-
6	-	-	-	-	7% [4]

Percent of subjects with bands and identification numbers of those subjects in whom bands reacting with the anti-pro-SP-B-antibodies CTERMB, NFLANK, CFLANK, NFPROX and displaced by the CTERMB, NFLANK, CFLANK, NFPROX peptides, or the anti-pro-SP-C-antibody NPRO-SP-C-C2 and displaced by the respective peptide, were identified. Differences in the frequency of bands of all the disease groups were evaluated by the Fisher exact test and those with a P ≤ 0.05 were indicated by an * for comparison with the healthy control group or by a ^+ ^for comparison with the disease control group, bronchitis (see table 2). The identification numbers of the patients are given in square brackets []. Numbers in bold indicate bands not identified by the CTERMB antibodies.

Among the PAP patients, only 2 had consistent pro-SP-C bands (Tab. 4). Subject PAP 08, a patient with a heterozygous *SFTPC *mutation and previously described in detail, had 3 bands, and subject PAP 04, in whom no SP-C mutation was detected, had one band at 6 kD [17]. Those 2 patients with the *SFTPC *mutation g.2125G>A [17] had no pro-SP-C bands with this antibody.

### Infants with chronic respiratory distress of unknown cause

**Figure 5:**
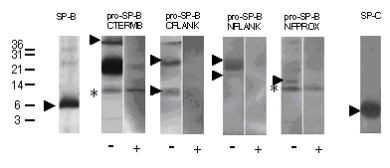
Children with chronic respiratory distress of unknown cause (cRD). Western blotting of a lavage from patient cRD 06 (NFLANK) and from patient cRD 07 (all other blots), performed as described in detail in the legend to figure 4. An asterisk marks non-specific bands, i.e. bands not displaced by the competing peptide. The arrowheads indicate the bands reacting with CTERMB at 40–42 kDa which are more frequently observed in these conditions than in the others. Similarly, with CFLANK, bands are seen at 40–42, 25–26, and 19–21 kDa. Cut off fragments likely generated during protein processing react with NFLANK or NFPROX. All bands were analyzed under reducing conditions.

The infants with chronic respiratory distress of unknown cause had no mutations of *SFTPB *or *SFTPC*, and normal SP-B and SP-C in their lavages (Tab. 1). Nevertheless, aberrant pro-SP-C was detected in one of these infants at 9 kDa (Tab. 5). Concerning the processing of pro-SP-B significant deviations from the pattern observed in the control groups were observed in some of these children with cRD. Indeed a pro-SP-B precursor at 40–42 kDa was observed more frequently in these patients (Fig. 5, Tab. 5). Similarly, as in PAP, bands reacting with NFPROX, representing fragments of the cleaved N-terminus, were detected (Tab. 5, Figs. 1 and 5).

**Table 5:** Pro-SP-B and pro-SP-C in 7 children with chronic respiratory distress of unknown cause (cRD)

	pro-SP-B				pro-SP-C
Detecting antibody	CTERMB	CFLANK	NFLANK	NFPROX	NPROSP-C-C2
M_r _of bands (kDa)					
40–42	57%* [2,5–7]	14% [**7**]	-	-	-
25–26	71% [2,3,5–7]	38%^+ ^[**4**,6,7]	57% [2,**4**,5,6]	-	-
19–21	-	-	14% [**6**]	14%§ [**6**]	-
15	-	-	-	29%^§ ^[**2,7**]	-
9	14% [6]	14% [**7**]	-	-	14% [6]
3.6	-	-	-	14%^§ ^[**3**]	-

Percent of subjects with bands and identification numbers of those subjects in whom bands reacting with the anti-pro-SP-B-antibodies CTERMB, NFLANK, CFLANK, NFPROX and displaced by the CTERMB, NFLANK, CFLANK, NFPROX peptides, or the anti-pro-SP-C-antibody NPRO-SP-C-C2 and displaced by the respective peptide, were identified. Differences in the frequency of bands of all the disease groups were evaluated by the Fisher exact test and those with a P < 0.05 were indicated by an * for comparison with the healthy control group or by a ^+ ^for comparison with the disease control group, bronchitis (see table 2). §indicates a significant difference to the disease control group, bronchitis, when all NFPROX reactive bands were combined (P < 0.01). The identification numbers of the patients are given in square brackets []. Numbers in bold indicate bands not identified by the CTERMB antibodies.

## Discussion

In this study we defined the presence and characteristics of SP-B, SP-C and their processing forms in bronchoalveolar lavages from children with severe chronic respiratory distress and in comparison groups of normal children and children with chronic obstructive bronchitis (Fig. 1). The major findings are the presence of mature SP-B and SP-C in all children, except those with SP-B deficiency, supporting the view that analysis of BAL for these surfactant proteins may aid in the diagnostic work up of children with severe respiratory distress. Overall pro-SP-C forms were rarely detected, and their presence was specific, but not pathognomonic for a SP-B deficiency due to *SFTPB *mutations. In addition, using epitope specific antisera, we identified unique pro-SP-B forms containing residues 145–160 of proSP-B (i.e. the "NFPROX" epitope) exclusively in BAL from patients with alveolar proteinosis and chronic respiratory distress. Taken together, the data suggest that immunobiochemical analysis of BAL can detect abnormalities in surfactant biosynthesis and metabolism associated with a variety of parenchymal lung diseases.

Of the 6 patients with SP-B deficiency defined as a lack of mature SP-B on Western blotting, 4 had mutations in *SFTPB *(Tab. 3). Based on our results, the biochemical analysis of BAL fluid for mature SP-B, previously thought to be diagnostic for SP-B deficiency, is not 100% specific, as there are additional cause(s) leading to a lack of SP-B. Possible mechanisms include mutations or secondary changes in regulatory elements or other defects in the synthesis and secretion of surfactant, as recently shown for the ABCA3 transporter [27].

An important finding of this study is the regular detection of certain pro-SP-B peptides in **BAL **from children without bronchoalveolar disease. Most prominent was a 25–26 kDa band, detected in almost all patients. This protein corresponds to removal of N'-terminal peptides from pro-SP-B, liberating 13–15 kDa fragments. SP-B is synthesized as a proprotein by alveolar type II epithelial cells and non-ciliated bronchiolar (Clara) cells; however, complete processing of the precursor to the biologically active, mature peptide occurs only in type II cells. Clara cells merely generate the 25 and 42 kDa precursors [28]. Thus, this intermediate represents a normal pro-SP-B processing intermediate of SP-B biosynthesis and could result from either constitutive secretion of this form by type II cells or from the physiologic release of 25 kD pro-SP-B into the airways by Clara cells. The 25–26 kD bands of pro-SP-B have previously been described in amniotic fluid from a 24-week-old human fetus, in lung tissue from an infant with severe bronchopulmonary dysplasia at the time of lung transplantation, as well as in normal adult lung tissue and lavages and plasma [21,29]. Here we show that these peptides are released into the bronchoalveolar space in normal patients. Since lamellar bodies do not contain pro-SP-B, this likely occurs via constitutive, non-regulated secretory pathways.

In children with pulmonary alveolar proteinosis we discovered increased amounts of a 19–21 kD intermediate which reacted against C-terminal pro-SP-B antisera and with the NFLANK SP-B antibody. This finding of a complex pro-SP-B intermediate containing both the C-terminal propeptide and a vestigial N-terminal propeptide (approximate residues 186–201) extends the work of Brasch and colleagues who also noted the presence of pro-SP-B forms containing C-terminal propeptide epitopes [30]. Consistent with our data, this group also found that, in contrast to patients with congenital respiratory distress due to SP-B deficiency, the appearance of pro-SP-C forms in these PAP patients was a rare occurrence. Thus, despite similar chest x-rays and histopathological findings, the BAL profile for SP-B, SP-C and their proforms appears useful in distinguishing PAP from SP-B deficiency of any etiology.

Children with chronic respiratory distress of unknown cause (cRD) exhibited the 40–42 kD proprotein with increased frequency. The N'-terminal peptides liberated from pro-SP-B pre-protein during intracellular processing, i.e. 13–15 kDa peptides or smaller fragments and reacting with NFPROX, were found exclusively in both cRD and PAP (Fig. 1, Tab. 4, Tab. 5). As such they may give diagnostic hints for the involvement of processing defects in, especially in pediatric PAP.

Other peptides reacted with the antibodies directed to the flanking aminoacids next to the SP-B core (NFLANK and CFLANK). The presence of these relatively rarely observed bands at 11 to 15 kDa was not related to specific clinical features of the subjects, i.e. more pronounced lung injury, high protein to phospholipids ratio or high abundance of SP-B. Both, a 9 kDa intermediate, reactive to NFLANK [21] and a 9 kDa band reacting with antibodies directed to the C'-terminal flanking of pro-SP-B, have previously been observed in human isolated type II cells and fetal lung. Such bands were indeed detected in the lavages we investigated, although very rarely.

Pro-SP-C peptides were never detected in the control groups. This is in agreement with an earlier observation on a limited number of samples [31]. However, we found pro-SP-C forms that were clearly, but not exclusively, associated with SP-B deficiency or *SFTPC *mutation. On the other hand, not all infants with *SFTPB *(496delC) or *SFTPC *(R167Q) mutations had pro-SP-C in their lavages. Thus the presence of pro-SP-C in lavages may give strong, but surely not definitive, diagnostic evidence for SP-B and SP-C processing defects.

The aberrant pro-SP-C species observed in patients with SP-B deficiency carrying the 121ins2 mutation consists of a N-terminal extension of SP-C by the N-flanking 12 aminoacids of pro-SP-C [13]. The pro-SP-C forms observed in patients not bearing a *SFTPB *mutation clearly differed in molecular weights from those detected in SP-B deficiency, suggesting that several processing defects may result in aberrant pro-SP-C in the alveolar space.

## Conclusion

Here we defined the presence and characteristics of SP-B, SP-C and their processing forms in bronchoalveolar lavage fluids from children with severe chronic respiratory distress and in comparison groups of normal children and children with chronic obstructive bronchitis. Pro-SP-B of 25–26 kD was commonly detected in all groups, suggesting that this form currently does not appear to be of great diagnostic value for processing defects. In contrast, pro-SP-B of 19–21 kD was increased in children with alveolar proteinosis while the cleaved flanking propeptides liberated during intracellular processing of pro-SP-B were exclusively found in these children and in chronic respirator distress of unknown cause. Furthermore, although identified at low frequency, pro-SP-C forms when present in the BAL suggest the presence of one of the parenchymal diseases studied in this report. Though often associated with mutations in SFTPB and SFTPC genes, this was not an exclusive finding limiting the usage of pro-SP-C as a surrogate for SFTP/SFTPC diagnostic screening procedures. Taken together, our results demonstrate that significant perturbations in the metabolism of these hydrophobic surfactant proteins occur in a variety of chronic lung diseases.

## Competing interests

The author(s) declare that they have no competing interests.

## Authors' contributions

MG designed the study, categorized and organized the subjects, wrote initial drafts of the manuscript, SS performed the blots, MT and MB determined the genotype of the patients, MG, SS, MS, AB, MT and MB collected the case histories, reviewed the subjects data and clinical courses, SG and MFB participated in the design for the methods to blot for the surfactant proteins, helped to organize the data and the results, and to prepare the manuscript. All authors read and approved the final manuscript.
